# Supplementary material for: Antibiotic Resistance in Acetic Acid Bacteria Originating from Vinegar
Source: Antibiotics (Basel). 2024 Jul 5;13(7):626. doi: 10.3390/antibiotics13070626 (PMC11274321; doi:10.3390/antibiotics13070626)
Supplement: Supplementary file 1 [file antibiotics-13-00626-s001.zip › antibiotics-3058888-supplementary.pdf]

Supplementary Table S1. Microbiological MIC values (µg/mL) of acetic acid bacteria against 10 antibiotics.

No.	Strain name	Microbiological MIC values (µg/mL) of Acetic acid bacteria against antibiotics (10 kinds)									
		Ampicillin	Chlolamphenicol	Erythromycin	Gentamicin	Streptomycin	Kanamycin	Clindamycin	Tetracycline	Aztreonam	Ciprofloxacin
		AM	CL	EM	GM	SM	KM	CM	TC	AT	CI
1	*K. swingsii* YU19	4	>256	>256	2	1.0	0.75	>256	0.50	>256	16
2	*K. xylinous* CV4	1.5	>256	>256	1.0	2	1.0	>256	1.5	>256	>32
3	*K. intermedius* CV2	0.75	>256	>256	6	1.0	1.0	>256	0.25	>256	3.0
4	*K. saccharivorans* CV1	1.5	>256	>256	1.0	0.75	0.25	>256	6	>256	>32
5	*A. malorum* CV11	2	>256	>256	1.5	2	0.75	>256	0.25	>256	>32
6	*A. cerevisiae* KSO5	24	128	>256	1.5	0.75	0.75	>256	1.5	>256	>32
7	*A. pasteurianus* A33	0.19	>256	192	8	2	0.75	>256	4	>256	>32
8	*A. pasteurianus* A37	1.5	>256	>256	6	2	0.75	>256	0.50	>256	>32
9	*A. pasteurianus* JGB20-11	0.75	>256	>256	4	3	2	>256	8	>256	>32
10	*A. pasteurianus* JGB21-17	16	96	>256	4	1.5	1.0	>256	4	>256	>32
11	*A. pasteurianus* JGB21-20	2	192	>256	0.75	0.75	0.19	>256	3	>256	>32
12	*A. pasteurianus* GHA7	2	192	32	1.0	0.5	0.19	>256	6	>256	>32
13	*A. pasteurianus* GHA20	2	192	32	1.5	0.5	0.19	>256	6	>256	32
14	*A. pasteurianus* GYA23	2	128	>256	1.0	1.5	0.5	>256	4	>256	12
15	*A. pasteurianus* GHG8	3	>256	>256	2	3	4	>256	6	>256	32
16	*A. pasteurianus* GHG17	4	>256	64	1.0	1.5	0.5	>256	8	>256	>32
17	*A. pasteurianus* A11-2	1.5	>256	3	6	0.75	0.5	>256	0.75	>256	>32
18	*A. pasteurianus* GSR2	1.0	>256	64	2	0.75	0.5	>256	2	>256	32
19	*A. pasteurianus* CHR1	1.0	>256	8	1.5	3	1.0	>256	6	>256	24
20	*A. pasteurianus* GAR12	0.75	128	128	1.0	1.5	0.75	>256	4	>256	12
21	*A. pasteurianus* GSB12	1.5	128	6	1.5	0.75	0.5	>256	6	>256	>32

22	*A. pasteurianus* GYO12	0.75	128	>256	4	1.5	1.5	>256	4	>256	24
23	*A. pasteurianus* A24	0.75	>256	6	1.5	2	1.0	>256	2	>256	24
24	*A. pasteurianus* JKR1	1.0	>256	>256	2	2	1.0	>256	1.5	>256	16
25	*A. pasteurianus* GSB8	1.0	128	4	1.0	1.0	0.38	>256	2	>256	24
26	*A. pasteurianus* GSB26	1.5	128	4	1.5	3	1.0	>256	3	>256	32

Spplementary Table S2. The putative proteins associated with antibiotic resistance mechanisms through the analysis of the *K. saccharivorans* CV1 genome using the COG database.

**Locus_Tag**	**Start**	**End**	**Strand**	**Type**	**Ref**	**GI**	**Product**	**COG_blast**	**Identity**	**e-value**	**bitscore**
CV1_1_00438	473492	475054	+	CDS	162147862	2E+08	multidrug transporter MurJ	Peptidoglycan biosynthesis protein MviN/MurJ, putative lipid II flippase	66.14	0	614
CV1_1_00646	710066	711985	-	CDS	162148117	2E+08	penicillin-binding protein 2	Cell division protein FtsI/penicillin-binding protein 2	80.31	0	1046
CV1_1_00714	788591	791716	-	CDS	162147080	2E+08	multidrug efflux RND transporter permease subunit	Multidrug efflux pump subunit AcrB	82.8	0	1764
CV1_1_00716	793056	793757	+	CDS	162147078	2E+08	TetR family transcriptional regulator	DNA-binding transcriptional regulator, AcrR family	43.19	1.00E-46	161
CV1_1_00988	1064032	1066596	-	CDS	162147176	2E+08	penicillin-binding protein 1A	Membrane carboxypeptidase/penicillin-binding protein	71.1	0	1141
CV1_1_01019	1100797	1102086	-	CDS	162147138	2E+08	Bcr/CflA family drug resistance efflux transporter	Predicted arabinose efflux permease, MFS family	75.82	0	625
CV1_1_01110	1194198	1195631	+	CDS	162147077	2E+08	multidrug transporter	Na+-driven multidrug efflux pump	69.85	0	628
CV1_1_01148	1229058	1230290	+	CDS	162146936	2E+08	Bcr/CflA family drug resistance efflux transporter	Predicted arabinose efflux permease, MFS family	63.04	8.00E-168	485
CV1_1_01285	1364599	1364931	+	CDS	560142401	6E+08	multidrug transporter	Multidrug transporter EmrE and related cation transporters	60.55	3.00E-37	129
CV1_1_01353	1425805	1426791	+	CDS	258542396	3E+08	multidrug export protein EmrA	Multidrug resistance efflux pump	53.25	1.00E-101	310
CV1_1_01354	1426788	1429568	+	CDS	258542397	3E+08	multidrug ABC transporter ATP-binding protein	ABC-type sugar transport system, ATPase component	66.96	0	1244
CV1_1_01527	1616539	1617129	+	CDS	15597776	2E+07	Modulator of drug activity B	Putative NADPH-quinone reductase (modulator of drug activity B)	64.8	3.00E-93	278
CV1_1_01529	1618799	1619986	-	CDS	162149611	2E+08	multidrug transporter	Multidrug efflux pump subunit AcrA (membrane-fusion protein)	60.88	3.00E-171	492
CV1_1_01812	1937261	1938547	+	CDS	258542015	3E+08	multidrug transporter	Multidrug efflux pump subunit AcrA (membrane-fusion protein)	62.87	0	553
CV1_1_01856	1973431	1976727	-	CDS	162149137	2E+08	acriflavine resistance protein B	Multidrug efflux pump subunit AcrB	76.86	0	1608
CV1_1_01857	1976724	1979900	-	CDS	162149136	2E+08	multidrug transporter	Multidrug efflux pump subunit AcrB	83.38	0	1744
CV1_1_01858	1979971	1981275	-	CDS	162149135	2E+08	multidrug transporter	Multidrug efflux pump subunit AcrA (membrane-fusion protein)	72.68	0	535

CV1_1_01859	1981394	1982869	-	CDS	158422726	2E+08	multidrug transporter	Predicted arabinose efflux permease, MFS family	48.48	1.00E-135	409
CV1_1_01872	1998233	1999342	+	CDS	162149123	2E+08	multidrug export protein EmrA	Multidrug resistance efflux pump	69.34	2.00E-171	491
CV1_1_02411	2543795	2545162	-	CDS	162147982	2E+08	Multidrug resistance protein NorM	Na+-driven multidrug efflux pump	48.15	2.00E-104	326
CV1_1_02500	2648682	2649878	+	CDS	162149135	2E+08	multidrug transporter	Multidrug efflux pump subunit AcrA (membrane-fusion protein)	65.3	7.00E-165	478
CV1_1_02501	2649878	2653048	+	CDS	162149136	2E+08	multidrug transporter	Multidrug efflux pump subunit AcrB	72.74	0	1507
CV1_2_00024	33155	34360	-	CDS	260752743	3E+08	multidrug transporter	Multidrug efflux pump subunit AcrA (membrane-fusion protein)	48.35	9.00E-111	338
CV1_2_00090	116619	117953	-	CDS	162149599	2E+08	Erythromycin esterase	Erythromycin esterase homolog	71.97	0	619
